# The Relationship between ALA16VAL Single Gene Polymorphism and Renal Cell Carcinoma

**DOI:** 10.1155/2014/932481

**Published:** 2014-01-23

**Authors:** Dogan Atilgan, Bekir S. Parlaktas, Nihat Uluocak, Engin Kolukcu, Fikret Erdemir, Huseyin Ozyurt, Unal Erkorkmaz

**Affiliations:** ^1^Department of Urology, Gaziosmanpasa University Medical Faculty, Tokat 60100, Turkey; ^2^Department of Biochemistry, Gaziosmanpasa University Medical Faculty, Tokat 60100, Turkey; ^3^Department of Biostatistics and Medical Informatics, Medical Faculty, Sakarya University, Turkey

## Abstract

*Objectives*. The aim of this study was to investigate the association of RCC and Ala16Val polymorphism in Turkish patients with RCC. *Materials and Methods*. A total of 41 patients with RCC who underwent radical or partial nephrectomy in our clinic and 50 healthy volunteers living in the same geographic area were included in this study. DNA samples from serum of RCC patients and controls were genotyped for MnSOD polymorphism analysis. Genotype ratios and allele frequencies were compared between two groups and odd ratios with 95% confidence intervals were calculated statistically. A *P* value of <0.05 was considered statistically significant. *Results*. There was a significant difference in the MnSOD genotype distributions between the RCC patients and the controls in terms of Ala/Ala+Ala/Val and Val/Val genotypes (*P* = 0.039). The Ala/Ala+Ala/Val genotypes were found significantly suspicious for RCC with an OR of 2.64 (95% CI = 1.06–6.69, *P* = 0.039). In addition, Ala allele was found significantly suspicious for RCC with an OR of 2.26 (95% CI = 1.24–4.12, *P* = 0.009). *Conclusion*. Our study indicated that MnSOD Ala16Val polymorphism may be one of the many genetic factors for renal cancer susceptibility in Turkish patients.

## 1. Introduction

Renal cell carcinoma (RCC) is the most common malignancy of the kidney and it constitutes approximately 3% of all adult malignancies and more than 90% of renal cancers [1]. RCC originates from the tubular structures of the kidney and is classified into 4 main histological types. Of all types, the most common type seen clear cell renal cell carcinoma (ccRCC) accounts for about 75% of all RCC cases [2]. Cigarette smoking, body mass index, and heredity are the most important risk factors that are associated with RCC. Approximately, 4% of all RCC are hereditary as well [3].

Single nucleotide polymorphism (SNP) is defined as a DNA sequence variation occurring when a single nucleotide, A, T, C, or G, in the genome differs between members of a biological species or paired chromosomes in a human [4]. The association of SNPs in genomes with various systemic diseases and malignancies has been shown in many previous studies. In addition to the SNPs in genes which provide production of superoxide dismutase (SOD), glutathione peroxidase (GPX), and paroxonase (PON), a wide range of human diseases like cancer, infectious diseases, autoimmune, neuropsychiatric, sickle-cell anemia, *β* Thalassemia, and cystic fibrosis may result due to SNPs [5–7]. As a result, the diseases which develop due to different SNPs may become relevant pharmacogenomic targets for drug therapy [8]. The SNPs without an observable impact on the phenotype are still useful as genetic markers in genome-wide association studies, because of their quantity and the stable inheritance over generations [9].

The enzyme SOD is one of the most important members of the antioxidant defence system and it converts superoxide free radical (O_2_) to hydrogen peroxide (H_2_O_2_) [10]. MnSOD Ala16Val genotype (rs4880 SNP, present in exon 2 and substitutes a C>T at position 2734 which changes the amino acid from alanine (Ala) to valine (Val) at position 16) has been investigated in numerous studies and associated with various systemic diseases and malignancies [11].

In this study, we aimed to investigate the association of RCC and Ala16Val polymorphism in Tokat region and according to our knowledge this is the first study in the literature which investigated Ala16 Val polymorphism in RCC.

## 2. Materials and Methods 

After the approval of the local ethical committee permission, a total of 41 (29 males and 12 females) patients with RCC and who under went radical or partial nephrectomy operations in our urology clinic were included in this study. The control group included 50 healthy volunteers (37 men and 13 women), chosen at the same time, who were free of any chronic diseases, having no history of any cancer, and living in the same geographic area. They were matched with cases in age and gender. Smoking status and body mass index (BMI) of cases and controls were also evaluated. Tumor type, Fuhrman grade and tumor stage of the RCC patients were examined. Approximately 5 cc of venous blood was taken from patients and controls for the determination of genotype.

### 2.1. Genotyping

Genomic DNA was extracted from the peripheral leukocytes of the collected EDTA-anti-coagulated blood by the High Pure PCR Template Preparation Kit (Roche Molecular Biochemicals, Mannheim, Germany) according to the manufacturer's instructions. To identify MnSOD Ala-9Val SNPs, genotyping was performed using PCR amplification and polymorphisms were detected with hybridization probes labeled with fluorescent dyes (LightCycler 480 II Real-Time PCR System, Roche Diagnostics, Mannheim, Germany). Target fragments of the human MnSOD genes were amplified with specific primers. To detect the MnSOD Ala-9Val polymorphism, we applied 10 pmol of the forward primer 50-CAGCCTGCGTAGACGGTCCC-30 and the reverse primer 50 CGTGGTGCTTGCTGTGGTGC-30, and 3 pmol of the sensor probe 50 CTCCGGCTTTGGGGTATCTG-fluorescein-30 and the anchor probe 50-LC Red 640 GCTCCAGGCAGAAGCACAGCCTCCp-30. The LC FastStart Master Hybridization Probes buffer (Roche Diagnostics Inc.) was used as a reaction buffer. All primers and hybridization probes were designed and synthesized by TIB MOLBIOL (Berlin, Germany). The genotypes were identified by running a melting curve with specific melting points (Tm). Wild type MnSOD Ala exhibits a Tm of 65 ± 0.5°C. The allele variant MnSOD Val exhibits a Tm of 56 ± 0.5°C. The PCR reaction was as follows: initial denaturation at 95°C for 10 min, followed by 20 cycles at 95°C for 10 s, and annealing at 60°C. And then a melting curve was recorded by an initial increase in temperature to 95°C, cooling the reaction mixture to 40°C at 20°C/s, holding for 30 s, and then slowly heating it to 85°C at 0.1°C/s with continuous acquisition. Finally, the fluorescence signal was plotted against temperature in real time to produce melting curves for each sample.

### 2.2. Statistical Analysis

Pearson's, Yates corrected, and Fisher's Chi-Square test were used to compare the genotypes and other categorical data between control and case groups. All categorical data were presented as count and percentage. Two independent samples *t*-test was used to compare the continuous data (age and BMI). Continuous data were presented as mean ± standard deviation. A *P*  value<0.05 was considered significant. Analyses were performed using commercial software (IBM SPSS Statistics 20, SPSS Inc., an IBM Co., Somers, NY).

## 3. Results 

The characteristics of the patients and control groups were presented in Table 1. Mean age and female/male ratios were 62.18 ± 8.44 and 1/1.35 in control group and 59.54 ± 12.96 and 1/1.41 in patients group. Mean BMI was 26.02 ± 3.95 and smoking rate was 26% in control group while in RCC group these values were 25.54 ± 3.96 and 26.859%, respectively. There was no statistically significant difference between groups in terms of age, female/male ratio, BMI, and smoking status. Tumor types, Fuhrman grades, and clinical stage of the patients were presented in Table 2. Histologic subtyping revealed that there were 35 (85.4%) clear cell RCC, 2 (4.9%) papillary RCC, 2 (4.9%) chromophobe RCC, and 2 (4.9%) cystic RCC cases. Distribution of Mn-SOD (Ala16Val) polymorphisms in patients with renal cancer and controls was presented in Table 3. In the control group, the distributions of the MnSOD Ala16Val genotypes were consistent with the Hardy-Weinberg equilibrium (HWE). The distributions of the MnSOD genotypes in RCC patients were also consistent with the HWE. The Val/Val genotype was found in 46%, the Ala/Val genotype was found in 38%, and the Ala/Ala genotype was found in 16% of controls. In the RCC patients, the frequencies of the MnSOD genotypes were 24% for Val/Val, 42% for Ala/Val, and 34% for Ala/Ala. In addition Ala dominant genotypes (Ala/Ala+Ala/Val) were found to be 76% in RCC group and 54% in control group. There was a significant difference in the MnSOD genotype distributions between the RCC patients and the controls in terms of Ala/Ala+Ala/Val and Val/Val genotypes. The Ala/Ala+Ala/Val genotypes were found significantly suspicious for RCC with an OR of 2.64 (95% CI = 1.06–6.69, *P* = 0.039). In terms of allele frequencies, Ala allele frequency was found to be 55% and Val allele frequency was found to be 45% in RCC group. However Ala allele frequency was 35% and Val allele frequency was 65% in control group. Similarly, Ala allele was found significantly suspicious for RCC with an OR of 2.26 (95% CI = 1.24−4.12, *P* = 0.009).

## 4. Discussion

The etiology of renal cell carcinoma is still unclear. As in the other cancer types, oxidative stress may play a critical role in the development of RCC. The relationship between RCC and lipid peroxidation was shown in several reports [12, 13]. Additionally, Lusini et al. showed that cytoplasmic SOD and GPX activities were lower in RCC tissues than in normal tissues [14].

The inability of elimination of the ROS has been associated with the development of many diseases and various types of cancer as well. Three different isoforms of SOD have been identified. SOD1 contains copper (Cu) and zinc (Zn) and is located in cytoplasm. SOD2 contains manganese and is located in mitochondria. SOD2 is the only antioxidant enzyme known existing in the mitochondria. Mitochondrion is the most important accumulating area of the ROS that is produced during normal cellular metabolism. Because of this reason SOD2 (MnSOD) becomes more important in antioxidant defence mechanism. SOD3 or extracellular SOD also consists of Cu and Zn.

MnSOD is the most important enzyme that provide detoxification of the accumulated ROS in mitochondria and its gene is located at chromosome 6q25 [15]. Numerous studies have been identified that chromosome 6q25 has been deleted in many types of cancer. Therefore MnSOD has been accepted as a tumor suppressor gene by some authors [16–20]. In addition, it has been shown that oxidative stress may be associated with carcinogenesis and genetic variants in the genes encoding the enzymes related to ROS detoxification have been found to affect individual susceptibility to cancer development [21–23]. In this study, we investigated Ala9Val gene polymorphism and evaluated its effect on RCC occurrence in Turkish population. Relationship between the levels of mitochondrial H_2_O_2_-O_2_ and carcinogenesis has been demonstrated in several studies [24–26]. According to Sutton et al. Ala form is targeted into mitochondria. The different structures of the variants may affect interactions with the Tim 23 import channel within the inner membrane that causes a reduced import in mitochondrial matrix and lower activity of SOD2 for the Val16 variant [27]. Hence, it is expected that the Val form would be associated with higher cancer risk. Nevertheless, association of Val form of SOD2 with higher cancer was reported only in a very few studies [28–31].

On the contrary, there were a lot of studies in the literature that advocated the presence of Ala allele as a risk factor for cancer. The reason of this opposition is still unclear. It was known that superoxide radicals dismutated to H_2_O_2_-O_2_ and SOD2 accelerated this reaction. Dasgupta et al. showed that increase in intracellular production of H_2_O_2_ by SOD2 was associated with decreased sensitivity to tumor necrosis factor-*α* mediated apoptosis. Therefore, as in the case of Val form, reduction of the SOD2 to enter mitochondrial matrix inhibits dismutation of superoxides to H_2_O_2_. Decreased level of H_2_O_2_ may lead to increased rate of programmed cell death ratio. As a result, the death of defective cells may supress the process of cancer development. This may be the possible cause of the association between Val form and lower risk of cancer [32].

The relationship between Ala/Ala genotype and cancer risk has been shown in many studies. Landi et al. found that the Ala/Ala genotype was associated with increased risk of malignant pleural mesothelioma [33]. In a study with high number of patients Millikan et al. reported that women with the Ala/Ala genotype had a significantly increased risk of breast cancer [34]. The variant Ala allele was also associated with increased risk of breast cancer among premenopausal women with lower antioxidant consumption [35, 36]. The relationship between Ala9Val polymorphism and prostate cancer also has been investigated in many studies. Kang et al. reported that Ala/Ala and Ala/Val genotypes were associated with prostate cancer in Caucasians [37]. Similarly Ergen et al. determined that Ala/Ala genotype was significantly high in prostate cancer patients [38]. Additionally, Arsova-Sarafinovska et al. identified that the Ala/Ala genotype was associated with a greater risk of prostate cancer being diagnosed under the age of 65 years [39].

The association of Ala/Ala genotype and aggressive behavior of tumor has also been studied previously. Mikhak et al. reported that Ala/Ala allele was significantly associated with more aggressive form of prostate cancer in men with low lycopene status [40]. Similarly, Woodson et al. also noted that Ala/Ala genotype was associated with high grade tumor in prostate cancer patients [41]. Ala allele was also associated with gastric cancer and gastric precancerous lesions [42]. Our study showed a significant association between RCC risk and the MnSOD Ala16Val gene polymorphism.

In conclusion, although it was studied with a very limited number of patients, this study indicated that Mn-SOD Ala16Val polymorphism may be one of the many genetic factors for renal cancer susceptibility in Turkish patients. It should be noted that Mn-SOD Ala16Val polymorphism in different populations showed variations due to ethnicity and various environmental factors should be considered in different geographic areas; therefore this result needs to be verified with further studies having larger sample size.

## Figures and Tables

**Table 1:** Demographic data of the groups.

	Groups	
	Control (*n* = 50)	RCC (*n* = 41)
Gender		
Male	37 (74.0)	29 (70.7)
Female	13 (26.0)	12 (29.3)
Age	62,18 ± 8,44	59,54 ± 12,96
BMI	26,02 ± 3,95	25,54 ± 3,96
Smoking		
No	37 (74.0)	30 (73.2)
Yes	13 (26.0)	11 (26.8)

**Table 2:** Tumor characteristics of the RCC patients.

Pathology	
RCC	40 (97.6)
Cystic RCC	1 (2.4)
Tumor subtype	
Clear cell	35 (85.4)
Cystic	2 (4.9)
Chromophobe	2 (4.9)
Papillary	2 (4.9)
Mean tumor size	6.00 ± 3,36
Fuhrman scores	
1	11 (26.8)
2	21 (51.2)
3	6 (14.6)
4	3 (7.3)
Stage	
1	27 (65.9)
2	9 (22.0)
3	4 (9.8)
4	1 (2.4)

**Table 3:** Distribution of Mn-SOD (Ala16Val) polymorphism in patients with RCC and controls.

Mn-SOD	Patients *n* = 41	Control *n* = 50	*P*	OR (95% CI)
Mn-SOD			0.05	
ALA16VAL				
Ala/Ala	14 [34%]	8 [16%]		
Ala/Val	17 [42%]	19 [38%]		
Val/Val	10 [24%]	23 [46%]		
(Ala/Ala + Ala/val)	31 [76%]	27 [54%]	0.039*	2.64 (1.06–6.69)
Val/Val	10 [24%]	23 [46%]		
Allele frequency			0.009*	2.26 (1.24–4.12)
Ala	45 [55%]	35 [35%]		
Val	37 [45%]	65 [65%]		

*There was statistically significant difference between groups.
